# Alcohol-dysregulated microRNAs in hepatitis B virus-related hepatocellular carcinoma

**DOI:** 10.1371/journal.pone.0178547

**Published:** 2017-05-31

**Authors:** Hao Zheng, Angela E. Zou, Maarouf A. Saad, Xiao Qi Wang, James G. Kwok, Avinaash Korrapati, Pinxue Li, Tatiana Kisseleva, Jessica Wang-Rodriguez, Weg M. Ongkeko

**Affiliations:** 1 Department of Surgery, University of California, San Diego, La Jolla, California, United States of America; 2 School of medicine, Yale University, New Haven, Connecticut, United States of America; 3 Department of Surgery, The University of Hong Kong, Hong Kong, China; 4 Department of Pathology, Veterans Administration Medical Center, San Diego, La Jolla, California, United States of America; 5 Department of Pathology, University of California, San Diego, La Jolla, California, United States of America; Saint Louis University, UNITED STATES

## Abstract

Alcohol consumption and chronic hepatitis B virus (HBV) infection are two well-established risk factors for Hepatocellular carcinoma (HCC); however, there remains a limited understanding of the molecular pathway behind the pathogenesis and progression behind HCC, and how alcohol promotes carcinogenesis in the context of HBV+ HCC. Using next-generation sequencing data from 130 HCC patients and 50 normal liver tissues, we identified a panel of microRNAs that are significantly dysregulated by alcohol consumption in HBV+ patients. In particular, two microRNAs, miR-944 and miR-223-3p, showed remarkable correlation with clinical indication and genomic alterations. We confirmed the dysregulation of these two microRNAs in liver cell lines treated by alcohol and acetaldehyde, and showed that manipulation of miR-223-3p and miR-944 expression induces significant changes in cellular proliferation, sensitivity to doxorubicin, and the expression of both direct-binding and downstream mRNA targets. Together, the results of this study suggest that alcohol consumption in HBV+ HCCs regulates microRNAs that likely play previously uncharacterized roles in the alcohol-associated carcinogenesis of HCC, and future studies of these microRNAs may be valuable for furthering the understanding and treatment of alcohol and HBV-associated HCC.

## Introduction

Hepatocellular carcinoma (HCC) is the most common form of liver cancer and the fifth most deadly cancer, affecting over 500,000 people worldwide every year [1, 2]. One well-established risk factor for HCC is chronic Hepatitis B (HBV) infection, which accounts for approximately 50% of all cases [3]. In the absence of diagnostic markers for early detection of the disease, HBV-related HCC exhibits extremely poor prognosis, with median survival of less than 16 months [4]. Another well-established risk factor is excessive alcohol use. Studies have shown that alcohol consumption significantly increases the risk of HCC in HBV-related patients [5]. Multiple epidemiologic and pathologic studies have reported the synergism between alcohol and HBV infection in the context of HCC [6–8]. Despite the recent advancements in the knowledge of HCC and cancer in general, the molecular and transcriptional effects of ethanol exposure on HCC pathogenesis and progression, and its specific effects in HCC with chronic viral hepatitis B background, remain poorly characterized.

microRNAs (miRNAs) are a family of small non-coding RNAs that may significantly enhance understanding of the specific functional effects, regulatory dynamics, and diagnostic, prognostic and therapeutic considerations that may pertain to alcohol use in HBV+ HCCs [9, 10]. Discovered to modulate the expression of a broad array of protein-coding genes via posttranscriptional regulation, miRNAs have been intensively studied as critical players in the carcinogenesis in many cancers, including HCC [11]. Several microRNAs, including miR-26 and miR-122, have been found to be significantly associated with HCC progression and highly associated with patient survival [12, 13]. In 2008, Budhu et al. reported a 20-miRNA metastasis signature that could predict primary HCC with venous metastasis from metastasis-free solitary tumors (p<0.001) [14]. However, to date, there have been no etiology-specific studies focusing on the role of alcohol-regulated miRNAs in HCC pathogenesis and progression.

In this study, we sought to expand current understanding of the link between alcohol consumption and HCC, using next-generation RNA-sequencing data from 130 HBV+ HCC patients and 50 normal liver tissues. After performing a series of differential expression analyses, we identified a panel of alcohol-associated miRNAs and subsequently investigated these transcripts *in vitro*. Utilizing normal liver and established HCC cell cultures, we determined that these candidate miRNAs, including miR-223-3p and miR-944, are directly modulated in the presence of alcohol and acetaldehyde and are potentially involved in the early stages of malignant transformation. Additionally, we evaluated how manipulating the expression of miR-223-3p and miR-944 might induce changes in cellular proliferation, sensitivity to doxorubicin, and the expression of both direct-binding and downstream mRNA targets. The results of this study suggest that alcohol consumption in HBV+ HCCs regulates several miRNAs that likely play previously uncharacterized roles in the alcohol-associated carcinogenesis of HCC.

## Materials and methods

### miRNA-sequencing datasets and clinical data

Level 3-normalized miRNA expression datasets and clinical data for 130 HCC patients and 50 tumor-adjacent normal liver tissues were obtained on 12 April 2016 from The Cancer Genome Atlas (TCGA) (https://tcga-data.nci.nih.gov/tcga). Patients were separated into two cohorts based on reported alcohol use and hepatitis B status in their clinical history: (1) Hepatitis B-positive drinkers and (2) hepatitis B-positive nondrinkers. Patients with clinical history of hepatitis C, hemochromatosis, or non-alcoholic fatty liver disease were excluded from the analysis to minimize confounding variables.Clinical characteristics for HCC patients used in this study are provided in S1 Table.

### miRNA differential expression analyses

miRNA read counts were extracted from the TCGA Level 3 gene expression files. The read count tables were imported into edgeR v3.0 (http://www.bioconductor.org/packages/release/bioc/html/edgeR.html) [15], and lowly expressed miRNAs (counts-per-million < 1 in more than one-half of samples) were filtered from the analysis. Following TMM normalization, pairwise designs were applied to identify significantly differentially expressed miRNAs in (1) HCC tumors from HBV+ drinkers versus normal liver tissue, (2) HCC tumors from HBV+ HCC drinkers versus HCC tumors from HBV+ non-drinkers, and (3) HCC tumors from HBV+ non-drinkers versus normal liver tissue. All miRNAs identified as differentially expressed in each of the three edgeR comparisons were retained as candidates.

### Association of candidate miRNAs with patient survival and somatic mutations

Survival analyses were performed using the Kaplan-Meier Model, with miRNA expression in HCC tumors designated as a binary variable based on expression above or below the median. Mutation calls for the HCC tumors were obtained from mutation annotation files (maf) generated by the Broad Institute GDAC Firehose on 5 September 2016. We focused our analysis on the 10 most frequently mutated genes in HCCs, as determined by Debuire et al. [16]. Wilcoxon rank sum tests were employed to test for significant associations between miRNA expression level (counts-per-million) and mutational status, as well as correlation with residual tumor status.

### Cell culture and treatments with ethanol and acetaldehyde

The non-cancerous liver cell line L02 as well as the human hepatoma cell line Hep3B were gifts from the Wang lab at University of Hong Kong. The cells were cultured in DMEM supplemented with 10% fetal bovine serum, 2% penicillin/streptomycin, and 2% L-glutamate (GIBCO) and maintained at 37°C in a humidified 5% CO_2_/95% air atmosphere. These cells were either exposed to ethanol for 7 days, or to acetaldehyde (Alfa Aesar) for 48 hours. The dose used for ethanol treatment was chosen to be 0.1% by volume (17 mM) to represent social drinking habits, as 0.1% is the blood alcohol level constituting legal intoxication in the U.S. [17]. For all ethanol-culture experiments, treatment media was replaced every 24 hours with fresh media containing the stated ethanol concentration. The tissue culture plates were also sealed with paraffin film to reduce evaporative loss of ethanol from the media. It has been shown that sealed culture vessels are able to maintain ethanol concentrations over significantly longer incubation periods [18]. A concentration of 50 μM of acetaldehyde was used in acetaldehyde treatment to represent a range of light to heavy drinking as determined by the saliva concentrations of alcohol consumers [19]. To account for the short evaporation half-life of acetaldehyde, treatments were performed every 4 hours and the tissue culture plates were also sealed with paraffin film.

### Quantification of miRNA expression by qRT-PCR

Total RNA was isolated (Fisher Scientific) from cultured cells following treatment with ethanol or acetaldehyde. cDNA was synthesized using the QuantiMiR^™^ RT kit (System Biosciences) as per the manufacturer’s instructions. Real-time PCR reaction mixes were created using FastStart Universal SYBR Green Master Mix (Roche Diagnostics), and run on a StepOnePlus^™^ Real-Time PCR System (Applied Biosystems) using the following program: 50°C for 2 min, 95°C for 10 min, 95°C for 30 s, and 60°C for 1 min, for 40 cycles. Relative expressions of mRNAs and miRNAs were calculated based on the RQ = 2^-ddCt method. U6 primers and a Universal Reverse Primer were used from the QuantiMiR^™^ RT kit, and custom primers (Integrated DNA Technologies) were ordered using the sequences listed in S2 Table.

### Knockdown of miR-944 and miR-223-3p

L02 and Hep3B were transiently transfected with miR-944 and miR-223-3p inhibitors (Integrated DNA Technologies) or control scramble sequences, using Lipofectamine 2000 (Invitrogen, Carlsbad, CA) according to manufacturer’s specifications.

### AlamarBlue viability assay

L02 and Hep3B cells were plated in 96-well flat-bottom tissue culture plates (Falcon) at a density of 5,000 cells per well. Cells were transfected with the miRNA inhibitors 24 hours after plating. After a 48-hour incubation period, cell viability was measured using an alamarBlue Cell Viability Assay (ThermoFisher) in accordance with the manufacturer’s protocol. Fluorescence was measured using a SafireII plate reader (Tecan) at a wavelength of 590 nm.

### MTS cell proliferation assays

L02 and Hep3B cells were plated in 96-well flat-bottom tissue culture plates (Falcon) at a density of 5,000 cells per well. For acetaldehyde experiments, cells underwent acetaldehyde treatment as previously described, following a 24-hour plating period. At the 36-hour acetaldehyde treatment time point, cells were transfected with the miRNA inhibitors. Cellular proliferation was analyzed using an MTS proliferation assay (Promega) in accordance with the manufacturer’s protocol, beginning 24 hours after the last acetaldehyde treatment.

For doxorubicin sensitivity experiments, cells were transfected with the miRNA inhibitors after a 24-hour plating period. They were subsequently exposed to one of several doses of doxorubicin ranging from 0–4 μg/mL [20, 21]. After a 48-hour incubation period, cellular proliferation was analyzed using an MTS proliferation assay (Promega). All assays were performed in triplicate wells and experiments were individually performed at least twice.

### g-H2AX Immunofluorescence

L02 and Hep3B were transfected with miR-944 and miR-223-3p inhibitors as previously described and cultured on coverslips. Cells were subsequently treated with 0.25 μg/mL doxorubicin for 48 hours, and were fixed with 4% paraformaldehyde and blocked in goat serum at room temperature, followed by incubation with anti-phospho-Histone H2AX (JBW301) mouse monoclonal antibody (Cell Signaling Technology). Cells were then incubated with Alexa Fluor 594 donkey anti-mouse secondary antibody (Life Technologies) and counterstained with Hoechst. Immunofluorescence images were obtained at 40X using an inverted fluorescence microscope.

### g-H2AX foci scoring

g-H2AX foci were quantified using the CellProfiler program (http://cellprofiler.org/). For each sample, the average number of g-H2AX foci per cell was determined using at least 3 images taken from different fields on the coverslip (containing ≥60 nuclei total). Statistical significance was assessed using Student’s t-test.

### Identification and qRT-PCR verification of miR-944 and miR-223-3p target genes

Putative mRNA targets of miR-944 and miR-223-3p were selected using the TargetScan (v6.2) (http://www.targetscan.org/), miRanda (v15.0) (http://www.microrna.org/microrna/home.do), miRmap (v15.0) (http://mirmap.ezlab.org) and miRDB (v1.1) (http://mirdb.org/miRDB/) target prediction algorithms based on miRNA seed region complementarity in the mRNA 3’UTR.

Total RNA was collected from L02 and Hep3B 48 hours after transfection with miR-944 and miR-223-3p inhibitors. cDNA was synthesized using M-MLV Reverse Transcriptase (Promega) and qRT-PCRs were performed as previously described, with *GAPDH* serving as endogenous control. Primers for the target genes were custom synthesized (Eurofins MWG Operon) using the following sequences and their sequences are listed in S3 Table.

## Results

### Identification of miRNAs dysregulated by alcohol consumption in HBV patients

**Fig 1:**
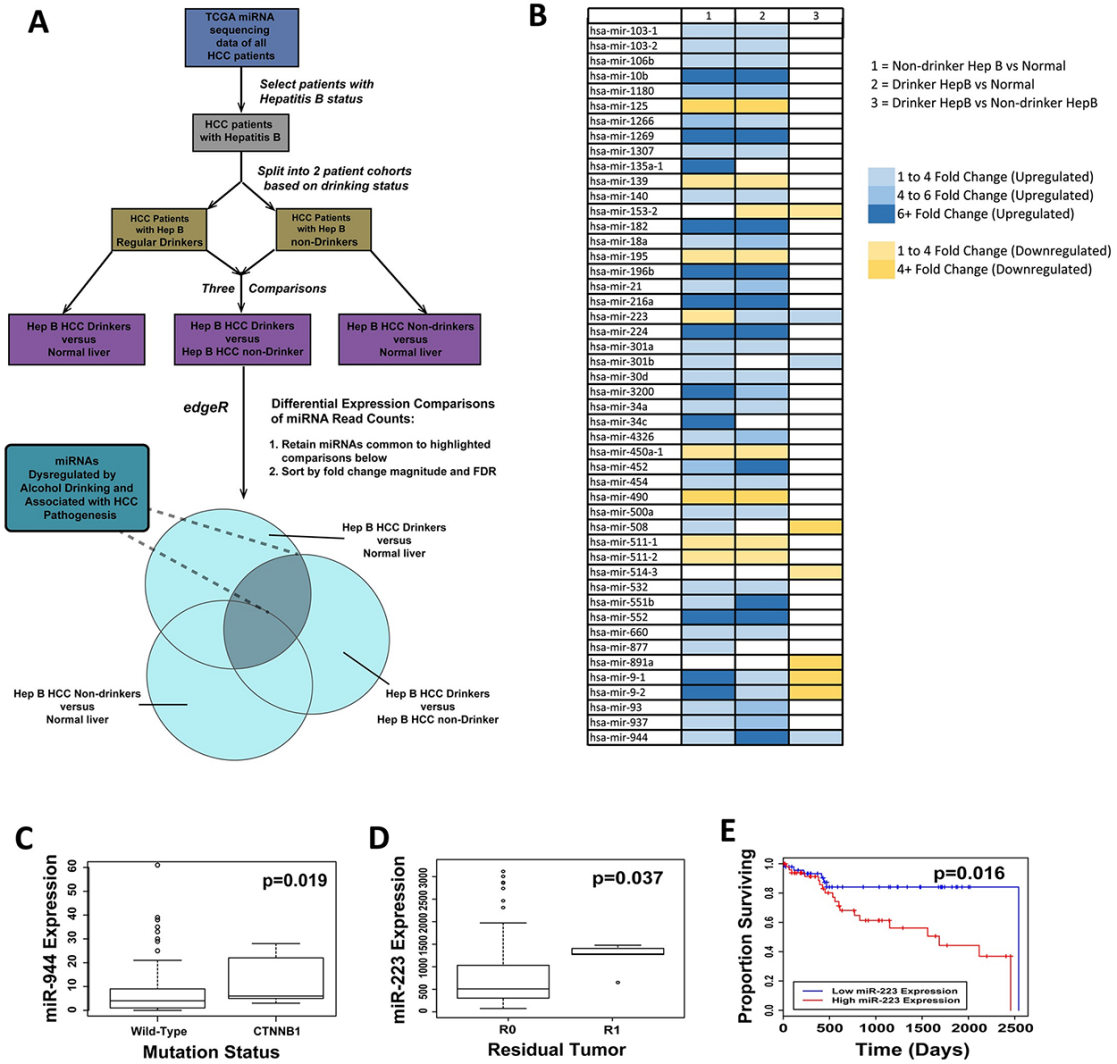
RNA-sequencing analysis identifies miRNAs that are dysregulated due to alcohol consumption in HBV-affected patients. (A) Schematic illustrating the analysis approach employed in this study. (B) Heat map depicting 48 miRNAs that are significantly dysregulated in at least two of the three comparisons (FDR < 0.05): nondrinker HBV+ vs normal, drinker HBV+ vs normal, drinker HBV+ vs non-drinker HBV+ (from left to right). (C) Boxplot showing significant correlation in expression level of miR-944 and somatic mutations of *CTNNB1* in HBV-associated HCC tumors. (D) Boxplot exhibiting significant correlation between miR-223 expression and patients’ tumor status following treatment. (E) Kaplan-Meier curves showing survival outcomes in HBV-affected HCC patients stratified by the expression level of miR-223.

We obtained clinical and microRNA-sequencing data containing normalized expression of 1,046 miRNAs for 130 HCC patients and 50 normal liver tissues from the TCGA database on 12 April 2016. In order to capture the miRNAs specifically dysregulated by alcohol in HBV patients, only patients with (1) HBV infection and (2) no history of conditions other than HBV and/or chronic alcohol consumption were selected. Using these patients, two cohorts were created: drinker HBV+ patients and non-drinker HBV+ patients. A total of three differential comparisons were performed: (1) HBV+ HCC drinkers versus normal liver; (2) HBV+ HCC drinkers versus HBV+ HCC non-drinkers; (3) and HBV+ HCC non-drinkers versus normal liver (Fig 1A). Using negative binomial-based differential expression testing, we identified a panel of 48 miRNAs that are significantly dysregulated between HBV+ drinker and HBV+ nondrinker HCC tumors. In particular, 5 miRNAs (miR-9-1, miR-9-2, miR-223, miR-944, and miR-153) were found to be significantly dysregulated (Fig 1B, Table 1), establishing themselves as associated with both alcohol drinking status and HCC tumor vs. adjacent normal tissue status. Among these, 2 miRNAs (miR-223 and miR-944) also showed strong correlation with genomic alterations and clinical indications associated with HCC. Notably, miR-944 expression significantly correlated with incidence of mutated *CTNNB1* (p = 0.019) (Fig 1C), a well-studied proto-oncogene [22], in HCC tumors; and miR-223 expression significantly correlated with presence of residual tumor after treatment (p = 0.037) (Fig 1D) and with univariate patient survival (p = 0.016) (Fig 1E).

**Table 1:** List of microRNAs that are significantly dysregulated in HBV+ HCC drinkers.

**Drinker HCC versus Non-drinkers HCC**			
	Fold change	logCPM	P values
***hsa-mir-223***	2.99	7.45	1E-08
***hsa-mir-9-1***	-4.54	9.20	2E-03
***hsa-mir-9-2***	-4.53	9.19	2E-03
***hsa-mir-153-2***	-3.03	3.59	2E-03
***hsa-mir-944***	3.12	1.51	4E-03
**Non-drinkers HCC versus Normal**			
	Fold change	logCPM	P values
***hsa-mir-223***	-1.80	7.31	1E-08
***hsa-mir-9-1***	13.03	8.99	3E-17
***hsa-mir-9-2***	12.95	8.99	3E-17
***hsa-mir-153-2***	1.38	3.74	1E-01
***hsa-mir-944***	2.84	1.06	1E-05
**Drinker HCC versus Normal**			
	Fold change	logCPM	P values
***hsa-mir-223***	1.77	8.22	6E-03
***hsa-mir-9-1***	3.42	6.85	1E-10
***hsa-mir-9-2***	3.39	6.84	1E-10
***hsa-mir-153-2***	-1.97	3.24	3E-06
***hsa-mir-944***	7.17	1.31	2E-11

### In vitro acetaldehyde and alcohol treatment validates alcohol-induced microRNA expression

**Fig 2:**
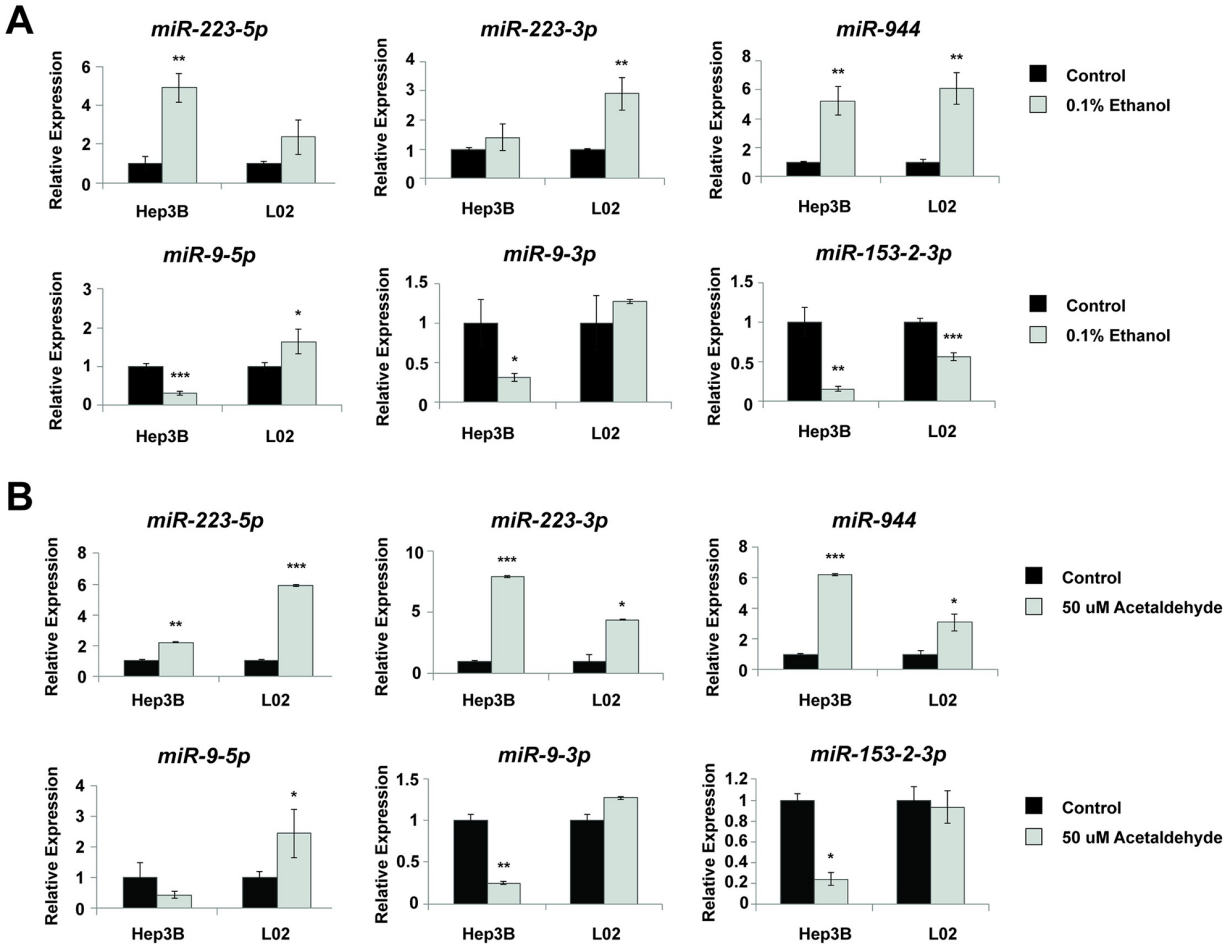
Ethanol and acetaldehyde treatment in liver cells verifies clinical data analysis in miRNAs expression. (A) qRT-PCR demonstrates that 7-day ethanol treatment dysregulates the expression level of 6 miRNAs in both Hep3B and L02. (B) qRT-PCR demonstrates that 48-hour acetaldehyde treatment dysregulates the same set of miRNAs in both cell lines. All bar graphs are presented as the mean and error bars representing standard deviations. *p<0.05, **p<0.01, ***p<0.001, Student’s t-test.

To validate that our selected miRNAs play a role in the alcohol-associated pathogenesis of HCC, we performed *in vitro* ethanol treatment using the normal liver cell line L02 and the HCC cell line Hep3B, derived from an HBV+ HCC patient. The cells were treated with 0.1% (17 mM) alcohol by volume once a day for 7 days, mimicking the blood alcohol level attained by social drinkers [18]. An MTS assay showed that there were no significant changes in cell survival in LO2 and Hep3B cells treated with ethanol (S1 Fig). Following alcohol treatment, two miRNAs (miR-223 and miR-944), previously identified to be overexpressed in HCC drinkers, were significantly upregulated in both L02 and Hep3B, with over 5-fold increase in miR-944 expression in both cell lines. Additionally, two miRNAs (miR-9 and miR-153-2-3p), previously identified to be downregulated in HCC drinkers, were inhibited in both cell lines upon alcohol treatment (Fig 2A).

Since ethanol metabolism by hepatocytes is thought to be the basis behind the pathogenesis of HCC in alcohol-consuming patients, we also treated our cell lines with acetaldehyde, the first and carcinogenic metabolite of ethanol. An MTS assay showed that there were no significant changes in cell survival in LO2 and Hep3B cells treated with acetaldehyde (S1 Fig). RT-qPCR revealed that when liver cell lines were exposed to physiologically relevant doses of acetaldehyde (50 uM) [19] continuously for 48 hours, significant dysregulation was observed in the expression of all four miRNAs (miR-223 and miR-944, miR-9, miR-153-2) (Fig 2B). Taken together, our data suggests that dysregulation of these miRNAs is due to alcohol metabolism, and may be an early event in the malignant transformation of liver cells, underscoring the importance of further investigating these miRNAs to better understand their roles in HCC.

### Knockdown of miR-944 and miR-223 decreases liver cell proliferation and mitigates the effect of acetaldehyde

**Fig 3:**
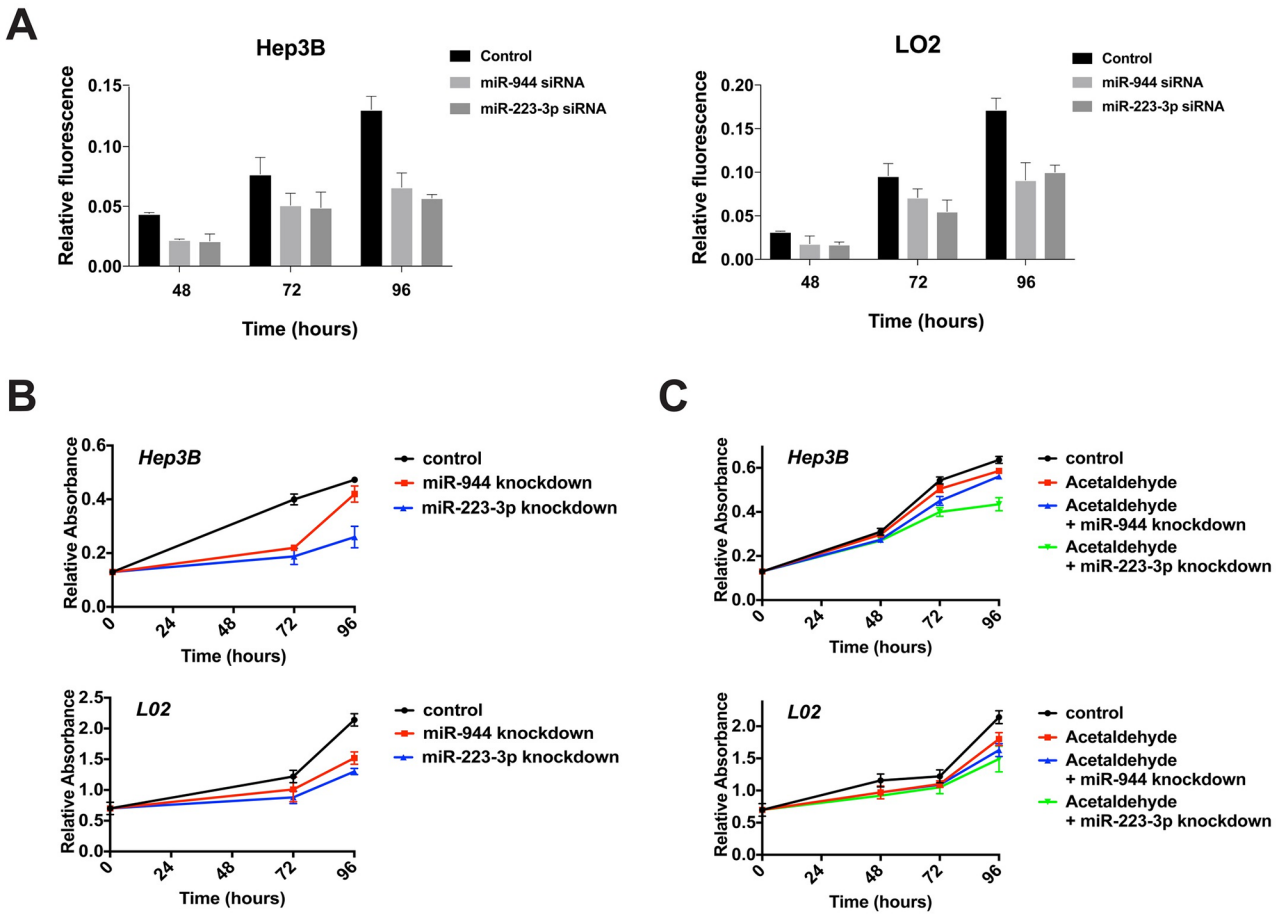
Inhibition of miR-944 and miR-223-3p inhibits cell viability and cell proliferation. (A) AlamarBlue cell viability assays evaluate the change in cell viability in L02 and Hep3B at 48, 72 and 96 hours after miR-944 and miR-223-3p knockdown. (B-C) Knockdown of miR-944 and miR-223-3p significantly inhibits cell proliferation in both the absence (B) and the presence (C) of acetaldehyde.

The two microRNAs, namely miR-223-3p and miR-944, were selected for further functional analysis as they showed the most consistent dysregulation in the sequencing analyses, correlation with clinical characteristics, and validated expression in vitro. miR-223-3p, instead of -5p, was chosen, as it was the more abundantly detected strand in the miRNA-sequencing data, and has been implicated in multiple studies in HCC and alcohol [23, 24]. To functionally characterize the effects of these two miRNAs, we investigated their influence on cell viability and cellular proliferation. Upon suppression of miR-223-3p and miR-944 using siRNAs, cell viability and cellular proliferation were significantly inhibited in both L02 and Hep3B within 96 hours, with nearly 2-fold decrease in both cell lines for miR-223-3p (Fig 3A and 3B). In addition, our results show that knockdown of the two miRNAs is capable of mitigating the effect of acetaldehyde by inhibiting cell growth. Acetaldehyde, being both carcinogenic and cytotoxic, was observed to inhibit cell growth at 50 uM in this study. When cells were treated with acetaldehyde and were simultaneously transfected with miRNA inhibitors, consistent decrease in cell proliferation was observed in both cell lines for both miRNAs compared to cells treated with acetaldehyde alone (Fig 3C).

### Inhibition of miR-223-3p and miR-944 sensitizes cells to doxorubicin-induced DNA damage and cell death

**Fig 4:**
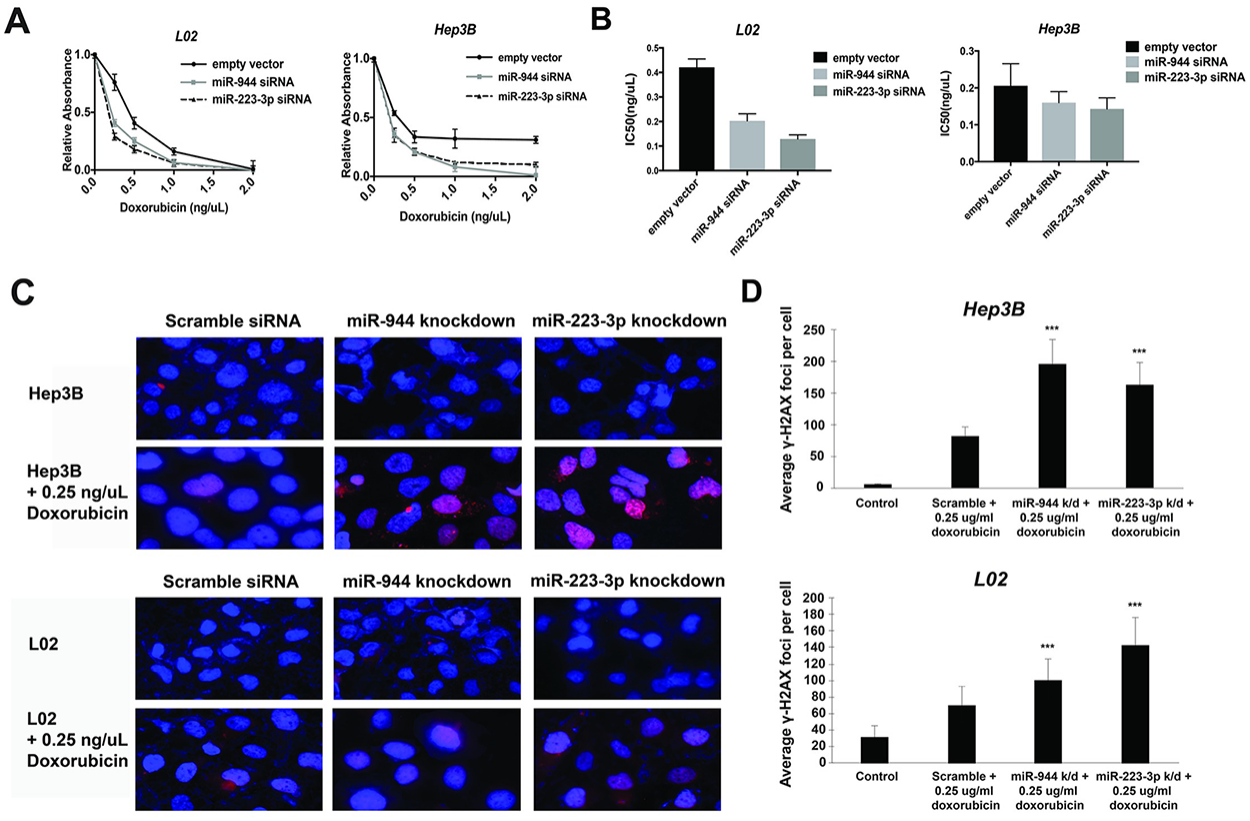
Inhibition of miR-944 and miR-223-3p enhances doxorubicin-induced cell death and DNA damage. (A-B) MTS assay indicates that miR-944 and miR-223-3p knockdown sensitizes liver cells to doxorubicin, as shown by the reduction in doxorubicin IC-50. (C-D) Immunofluorescence images showing increased expression of γ-H2AX in cells treated with both doxorubicin and miRNA siRNAs compared to cell treated with doxorubicin alone. Cells were stained with anti-γH2AX (red) and DAPI (blue).

To further explore the functional significance of miR-223-3p and miR-944, we tested their ability to sensitize cells to doxorubicin, a chemotherapy drug commonly utilized in treating HCC [25]. Our results indicate that knockdown of miR-223-3p and miR-944 promotes doxorubicin-mediated cell death (Fig 4A), with the IC50 for doxorubicin reduced by 52% and 71%, respectively, in L02. In Hep3B, while the difference in IC50 was modest, the effect of miRNA knockdown on cell death was remarkable at higher doses, where complete cell death was only achieved following miRNA knockdown (Fig 4B). Additionally, we assessed the effects of miR-223-3p and miR-944 on apoptotic DNA fragmentation at 0.25 ug/ml doxorubicin using the DNA double-strand break and apoptosis marker γ-H2AX. Immunofluorescence showed significant upregulation of punctate γ-H2AX foci in miR-223-3p and miR-944-knockdown cell lines treated with doxorubicin (Fig 4C), with miR-223-3p-knockdown doxorubicin-treated L02 and miR-944-knockdown doxorubicin-treated Hep3B exhibiting more than 2-fold induction of γ-H2AX foci compared to cells treated with doxorubicin alone (Fig 4D).

**Fig 5:**
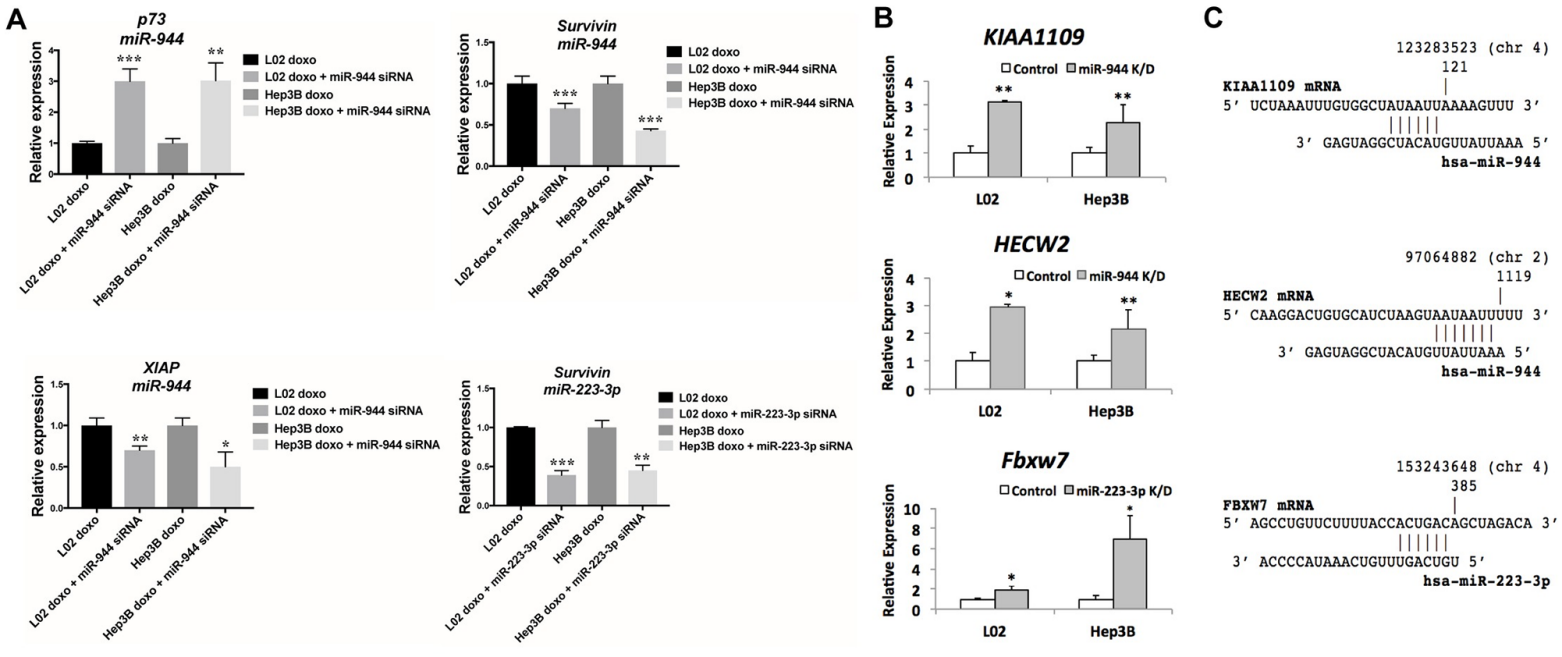
miR-944 and miR-223-3p knockdown in HCC cell lines upregulates predicted target tumor suppressor genes, and induces changes in key apoptosis gene expression. (A) Knockdown of miR-223-3p and miR-944 alters key apoptosis gene expression in Hep3B and L02. (B) Inhibition of miR-944 significantly upregulates both *HECW2* and *KIAA1109*; and inhibition of miR-223-3p significantly upregulates *Fbxw7*. (C) Putative seeding regions between the mature miRNAs and target mRNAs are presented.

Next, to further examine the increased sensitivity to doxorubicin imparted by miR-944 and miR-223-3p, we evaluated the expression of a panel of key apoptosis genes following miRNA knockdown at 0.25 ug/ml doxorubicin. Notably, p73 showed significant upregulation following miR-944 knockdown, with over 3-fold increase in expression in both cell lines. Apoptotic inhibitor Survivin, a direct target of Wnt pathway and a negative regulator of caspase, was found to be inhibited for miR-944 and miR-223-3p in both cell lines. Additionally, XIAP, another apoptotic inhibitor gene, also showed strong downregulation following miR-944 knockdown (Fig 5A).

### Prediction and PCR validation of mRNA targets for miR-223-3p and miR-944

To further define molecular roles for miR-223-3p and miR-944, we utilized four different computational programs (miRDB, TargetScanHuman, miRanda and miRmap) in searching for the putative target for the two miRNAs [26–29]. To avoid bias generated by the individual algorithms, only the targets that were commonly proposed in all four of the programs were selected. To validate these targets, we evaluated the expression levels of these putative targets following knockdown of miR-944 and miR-223-3p down the miRNAs in liver cell lines. Inhibition of miR-223-3p induced significant upregulation of *Fbxw7*, a tumor suppressor that has been previously reported to correlate with HCC prognosis and found to be consistently reduced in HCC tumors and cell lines [30–32]. Moreover, inhibition of miR-944 significantly upregulated both *HECW2* and *KIAA1109* (Fig 5B). The putative seeding regions between microRNAs and mRNAs are depicted in Fig 5C.

## Discussion

Despite substantial evidence implicating alcohol use as both an independent risk factor and cofactor in HCC [33], there remains a limited understanding of how alcohol directly promotes HCC pathogenesis and progression, and how alcohol operates in conjunction with other risk factors such as HBV infection, the most prevalent etiological agent for HCC [3]. Previous studies, although identifying key enzymes and signaling pathways compromised by hepatic ethanol metabolism [34, 35], have only sparsely examined alcohol-induced changes at the gene expression and transcriptional levels as they relate to the development of HCC. Thus, in this study, we sought to enhance knowledge of the contribution of alcohol exposure to HCC, by identifying and functionally characterizing miRNAs regulated by alcohol in HBV+ HCCs.

We are the first to undertake a focused investigation of the expression patterns of alcohol-associated miRNAs in HCC. Through a series of RNA-sequencing analyses of 130 HBV+ HCC patients from the TCGA database, stratified by drinking status, we identified 5 miRNAs (miR-9-1, miR-9-2, miR-153-2, miR-223, and miR-944) differentially expressed by more than 2-fold in chronic alcohol drinkers with HBV+ HCC in comparison to nondrinkers with HBV+ HCC. Consistent with previous HCC miRNA profiling studies [36–38], miR-9-1 and miR-9-2 were overexpressed in HBV+ HCC tumors relative to normal tissue; however, miR-9-1 and miR-9-2 were significantly downregulated in tumors from HBV+ HCC drinkers relative to tumors from HBV+ HCC non-drinkers (Fig 1B and Table 1). A previous study in fetal neural stem cells revealed that miR-9 and other miRNAs may be subject to coordinate or even opposing regulation following concurrent exposure to ethanol and nicotine, resulting in greater disruption to miRNA regulatory networks [39]. Further exploration of whether a similar interplay exists in HCC between alcohol use and HBV infection, in the context of regulating miR-9 and the other alcohol-associated HCC miRNAs we have identified, may be merited. Additionally, we found that miR-153-2 is upregulated relative to normal liver in HBV+ HCC non-drinkers, but downregulated in HBV+ HCC drinkers (Fig 1B and Table 1). Previously, Xia et al. reported that miR-153 suppresses epithelial-mesenchymal transition in HCCs [40], while Hua et al. and Chen et al. reported that miR-153 activates Wnt/β-catenin [41] and promotes resistance to chemotherapy and small molecular kinase inhibitors in HCC [42]. Our finding that the divergent expression and regulation of miR-153 may be dictated by exposure to alcohol may provide a means of resolving these conflicting studies of miR-153 expression and function in HCCs. We also observed downregulation of miR-223 in non-drinker HBV+ HCCs, and upregulation of miR-223 in drinker HBV+ HCCs, relative to normal tissue (Fig 1B and Table 1), a result which may similarly contribute to our understanding of what has previously appeared to be discrepant patterns of miR-223 expression in HCCs [24, 43, 44]. Meanwhile, to our knowledge, we are the first to report upregulation of miR-944 in HCC, and in drinkers with HCC relative to nondrinkers (Fig 1B and Table 1), although previous studies of miR-944 in have demonstrated its consistent upregulation in cervical and breast cancers [45, 46]. Our identification of a novel panel of miRNAs in HCCs linked to chronic alcohol exposure, coupled with our analyses correlating miR-944 with mutations in Wnt-activator *CTNNB1* [22] and miR-223 with patient survival and presence of residual tumor (Fig 1C–1E), suggests that alcohol-mediated dysregulation of miRNAs in HBV+ HCC modulates central pathways and contributes to clinically relevant phenotypes in HCC patients. In addition to showing that HBV+ HCC tumors assume miRNA expression profiles distinguishable by drinking history, our analyses also provide a complement to existing studies profiling hepatitis-associated miRNAs in HCC [47–50] and hepatitis- and alcohol-regulated miRNAs in cirrhosis and fatty liver disease [51–53]. In this context, our study contributes to a composite understanding of HCC not only as a malignancy in isolation but as part of a lineage of chronic liver diseases.

We found miR-9, miR-153-2, miR-223, and miR-944 to be dysregulated in normal cell line L02 and HBV+ HCC cell line Hep3B following treatment with physiologically relevant doses of ethanol and acetaldehyde [19], demonstrating that altered expression of these miRNAs is indeed directly caused by alcohol exposure, and likely involved in the early stages of HBV+ hepatic carcinogenesis. As acetaldehyde treatment alone also proved sufficient to differentially regulate these miRNAs, our results further corroborate the premise that the metabolism of ethanol to acetaldehyde is at least partially responsible for alcohol-associated HCC [34, 35]. In particular, we found that siRNA-mediated knockdown of miR-944 and miR-223-3p significantly decreased proliferation of L02 and Hep3B (Fig 3B), as well as the proliferation of L02 and Hep3B treated with acetaldehyde (Fig 3C). This potential of miR-944 and miR-223-3p to regulate cellular proliferation under both conditions suggests that the suppression of these miRNAs may be utilizable in preventing or mitigating the specific effects of alcohol and acetaldehyde in hepatocytes. In addition, we observed that miR-944 and miR-223-3p knockdown significantly sensitized L02 and Hep3B to doxorubicin (Figs 4A, 4B and 5A). As evidenced by relative accumulation of γ-H2AX foci, miR-944 and miR-223-3p inhibition potentiated doxorubicin-induced DNA double-strand breaks in L02 and Hep3B (Fig 4C). Notably, suppression of both miRNAs in doxorubicin-treated L02 and Hep3B also reduced mRNA levels of Survivin, an apoptotic inhibitor extensively associated with HCC proliferation [54], progression [55], and patient prognosis [56] (Fig 5A). miR-944 knockdown additionally restored expression of p73 and suppressed expression of *XIAP* (Fig 5A). Collectively, these findings suggest that miR-944 and miR-223-3p may represent possible therapeutic candidates that can be leveraged to overcome chemoresistance in HCC; previously, miR-944 and miR-223 were found to regulate cisplatin resistance in breast cancer and gastric cancer, respectively [46, 57].

The genes we have identified to be putative targets of miR-944 and miR-223-3p in alcohol-associated HCC further substantiate our proposed functions for these miRNAs as well. miR-944 was found to negatively regulate *KIAA1109* (Fig 5B), a gene newly associated with cancer risk and found to be recurrently mutated in pan-cancer studies [58, 59]; as well as *HECW*2 (Fig 5B), a ubiquitin ligase established as a direct target of miR-944 in cervical tumors [45]. *HECW2* has also been reported to stabilize and enhance p73 transcriptional activity [60], consistent with our findings of p73 induction following miR-944 knockdown in doxorubicin-treated cell lines (Fig 5A). However, further research on the specific roles of *KIAA1109*, *HECW2*, and p73 in HCC is warranted. Meanwhile, miR-223-3p was found to target *Fbxw7* (Fig 5B), a cell cycle regulator that promotes c-Myc and cyclin E degradation [61], suggesting that the well-documented miR-223/*Fbxw7* axis [62, 63] is functionally active in HCC, in addition to modulating resistance to γ-secretase inhibitor treatment in leukemia [64] and resistance to cisplatin in gastric cancers [57]. In the context of HCC, *Fbxw7* has already been found to be significantly downregulated in tumors compared to normal liver, with both mRNA and protein expression correlating with tumor stage, histological grade and disease-free survival [32, 61]. Future studies examining with greater resolution the possible dependence of these pathways on alcohol exposure in HBV+ HCC, and their potential involvement as downstream effectors of miR-944 and miR-223-induced changes to chemoresistance and apoptotic response, promise to add a new layer of insight into the alcohol-associated miRNA regulatory dynamics of HCC.

## Supporting information

S1 Table Clinical characteristics of patient cohorts. Table outlines the clinical characteristics of nondrinker HBV+ and drinker HBV+ cohorts.
(PDF)

S2 Table microRNA primer sequences. Table lists the sequences for forward microRNA primers used in qRT-PCR assays.
(PDF)

S3 Table microRNA target primer sequences. Table lists the sequences for forward and reverse primers used in qRT-PCR assays in testing microRNA targets.
(PDF)

S1 Fig Change in cell survival in liver cells treated with alcohol and acetaldehyde. Bar graphs depict the change in cell survival in liver cells treated with 0.1% ethanol treatment for 7 days and 50μM acetaldehyde for 48 hours.
(PDF)
