# Supplementary material for: Alcohol-dysregulated microRNAs in hepatitis B virus-related hepatocellular carcinoma
Source: PLoS One. 2017 May 31;12(5):e0178547. doi: 10.1371/journal.pone.0178547 (PMC5451132; doi:10.1371/journal.pone.0178547)
Supplement: S1 Table — Table outlines the clinical characteristics of nondrinker HBV+ and drinker HBV+ cohorts. (PDF) [file pone.0178547.s001.pdf]

S1 Table

		nondrinker HBV+ cohort	drinker HBV+ cohort
**Tumor grade**	G1	7.4%	9.4%
	G2	37.9%	34.4%
	G3	47.4%	50.0%
	G4	7.4%	6.3%
**AJCC staging edition**	4th	1.0%	0.0%
	5th	7.4%	3.1%
	6th	44.2%	15.7%
	7th	47.4%	81.3%
**AJCC tumor pathologic (pT)**	T1	58.9%	65.6%
	T2	29.5%	15.6%
	T3	3.2%	3.1%
	T3a	7.4%	9.3%
**AJCC nodes pathologic (pN)**	N0	87.3%	87.5%
	NX	12.6%	12.5%
**AJCC metastasis pathologic (pM)**	M0	91.6%	90.6%
	MX	7.4%	9.4%
**AJCC pathologic tumor stage**	Stage I	58.1%	67.7%
	Stage II	29.0%	12.9%
	Stage IIIA	8.6%	12.9%
	Stage IIIB	0.0%	6.3%
	Stage IIIC	2.2%	0.0%
	Stage IV	1.1%	0.0%
