# Supplementary material for: Alcohol-dysregulated microRNAs in hepatitis B virus-related hepatocellular carcinoma
Source: PLoS One. 2017 May 31;12(5):e0178547. doi: 10.1371/journal.pone.0178547 (PMC5451132; doi:10.1371/journal.pone.0178547)
Supplement: S2 Table — Table lists the sequences for forward microRNA primers used in qRT-PCR assays. (PDF) [file pone.0178547.s002.pdf]

S2 Table

	Sequence (5'-3')
hsa-miR-223-5p	CGTGTATTTGACAAGCTGAGTT
hsa-miR-223-3p	TGTCAGTTTGTCAAATACCCCA
hsa-miR-9-5p	TCTTTGGTTATCTAGCTGTATGA
hsa-miR-9-3p	ATAAAGCTAGATAACCGAAAG T
hsa-miR-153-2-3p	TTGCATAGTCACAAAAGTGAT C
hsa-miR-944	AAATTATTGTACATCGGATGAG
