# Supplementary material for: Alcohol-dysregulated microRNAs in hepatitis B virus-related hepatocellular carcinoma
Source: PLoS One. 2017 May 31;12(5):e0178547. doi: 10.1371/journal.pone.0178547 (PMC5451132; doi:10.1371/journal.pone.0178547)
Supplement: S3 Table — Table lists the sequences for forward and reverse primers used in qRT-PCR assays in testing microRNA targets. (PDF) [file pone.0178547.s003.pdf]

S3 Table

	Forward (5'-3')	Reverse (5' – 3')
KIAA1109	TAACTATCAGCAGCGACCCC	TGGTGCTGTGACCTTAGCTG
HECW2	AGTGGACCTGCAGAGCTTTC	GCCCAGGCTCAATTCTCCAT
Fbxw7	GGTCAGGACATTTGGTAGGGG	AAAGAGCGGACCTCAGAACC
P73	CGAAAATGCCAACAAACGGC	GTGTTGGAGGGGATGACAGG
XIAP	ATTTCCAGATTGGGGCTCGG	TTTGTAGACTGCGTGGCACT
Survivin	ACGACCCCATGCAAAGGAAA	CTGGTAAGCCCGGGAATCAA
