# Supplementary figures and images for: Alcohol-dysregulated microRNAs in hepatitis B virus-related hepatocellular carcinoma
Source: PLoS One. 2017 May 31;12(5):e0178547. doi: 10.1371/journal.pone.0178547 (PMC5451132; doi:10.1371/journal.pone.0178547)

**S1 Fig**

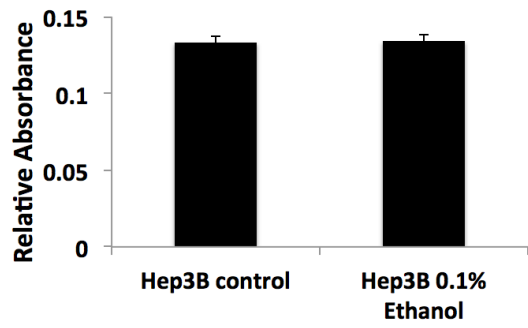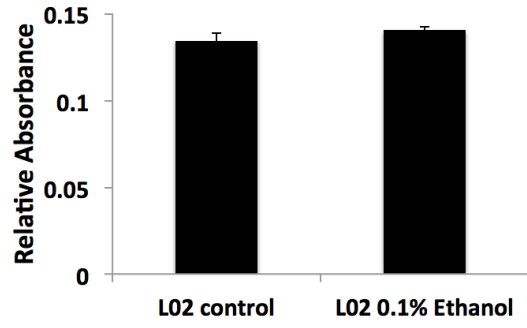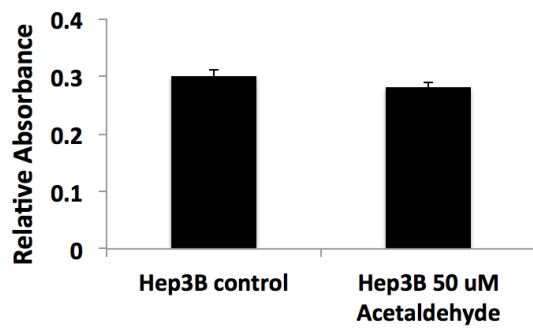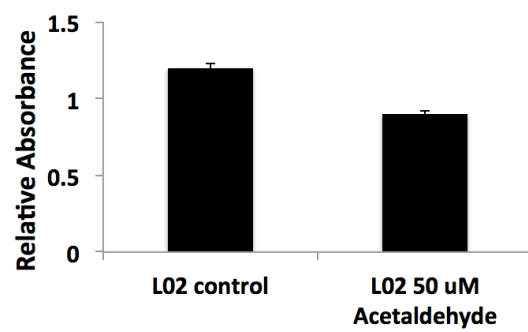

Supplement: S1 Fig — Bar graphs depict the change in cell survival in liver cells treated with 0.1% ethanol treatment for 7 days and 50μM acetaldehyde for 48 hours. (PDF) [file pone.0178547.s004.pdf]
